# Neuroprotective Effects of Jitai Tablet, a Traditional Chinese Medicine, on the MPTP-Induced Acute Model of Parkinson's Disease: Involvement of the Dopamine System

**DOI:** 10.1155/2014/542383

**Published:** 2014-04-02

**Authors:** Jia Liu, Jinlong Gao, Shaoang Tu, Shasha Xu, Ying Liu, Weihu Shang, Chenxin Gu, Yiyun Huang, Mei Han

**Affiliations:** ^1^College of Chemistry, Beijing Normal University, Beijing 100875, China; ^2^Yale PET Center, Department of Diagnostic Radiology, Yale University School of Medicine, New Haven, CT 06510, USA; ^3^Key Laboratory of Radiopharmaceuticals, Ministry of Education, College of Chemistry, Beijing Normal University, Beijing 100875, China

## Abstract

Jitai tablet (JTT) is a traditional Chinese medicine used to treat neuropsychiatric disorders. We previously demonstrated that JTT treatment led to increased level of dopamine transporter (DAT) in the striatum, thus indicating that JTT might have therapeutic potential for Parkinson's disease (PD), which is characterized by dysregulated dopamine (DA) transmission and decreased striatal DAT expression. The aim of this study was to investigate the neuroprotective effect of JTT on MPTP-induced PD mice. Using locomotor activity test and rotarod test, we evaluated the effects of JTT (0.50, 0.15, or 0.05 g/kg) on MPTP-induced behavioral impairments. Tyrosine hydroxylase TH-positive neurons in the substantia nigra and DAT and dopamine D_2_ receptor (D_2_R) levels in the striatum were detected by immunohistochemical staining and/or autoradiography. Levels of DA and its metabolites were determined by HPLC. In MPTP-treated mice, behavioral impairments were alleviated by JTT treatment. Moreover, JTT protected against impairment of TH-positive neurons and attenuated the MPTP-induced decreases in DAT and D_2_R. Finally, high dose of JTT (0.50 g/kg) inhibited the MPTP-induced increase in DA metabolism rate. Taken together, results from our present study provide evidence that JTT offers neuroprotective effects against the neurotoxicity of MPTP and thus might be a potential treatment for PD.

## 1. Introduction

Parkinson's disease (PD) is a debilitating chronic and progressive neurodegenerative disease. Between 0.5 and 1% of the population aged 65–69 years and 1–3% of those over 80 years old suffer from PD worldwide [1]. The pathophysiology of PD results from the progressive and selective loss of dopaminergic neurons, which leads to the depletion of striatal dopamine (DA), dopamine transporter (DAT), and DA receptors levels. PD patients exhibit several major clinical symptoms, such as bradykinesia, muscle rigidity, and resting tremor [2]. It has been reported that clinical symptoms appeared when approximately 80% of the striatal DA is lost [3]. Therefore, a major treatment strategy for PD focuses on DA supplementation, through the use of DA precursors such as levodopa (L-DOPA) [4], DA receptor agonists, and monoamine oxidase B (MAO-B) inhibitors [1, 5]. Madopar, which is composed of the DA precursor L-DOPA and the decarboxylase inhibitor benserazide, is commonly used to treat PD by increasing DA concentrations. Although it is used for the relief of PD motor symptoms, it does not prevent or slow the progressive degeneration of neuronal cells and its long-term use may induce various side-effects including fluctuations and abnormalities in involuntary movements [6]. DA receptor agonists and MAO-B inhibitors are both associated with the same issues, which greatly limit their clinical utility [1]. Thus, there is still an urgent need for efficacious therapies that not only act on the motor symptoms of PD but also protect neuronal cells and present low side-effect profiles.

Traditional Chinese medicine (TCM) may offer therapies with better efficacy and fewer side-effects [7]. TCM preparations usually contain more than one component and target multiple body systems for maximal therapeutic effects [7, 8]. As such, TCM may prove superior to other pharmacotherapies in the treatment of chronic and degenerative diseases such as PD [8]. The Jitai tablet (JTT) is a traditional herbal formula consisting of the following ingredients:* Papaveraceae Corydalis (yan hu suo), *10.20%;* Solanaceae Datura metel (yang jin hua),* 2.18%;* Lamiaceae Salvia Miltiorrhizae (Dan shen),* 16.87%;* Araliaceae Panax ginseng (Ren shen),* 2.18%;* Apiaceae Angelica sinensis (Dang gui),* 10.20%;* Apiaceae Ligusticum chuanxiong (Chuan xiong),* 5.71%;* Asteraceae Carthamus tinctorius (Hong hua),* 10.20%;* Ranunculaceae Aconitum (Fu zi),* 2.18%;* Myristicaceae Myristica cagayanensis (Dou kou),* 2.18%;* Asteraceae Aucklandia (Mu xiang),* 5.71%;* Thymelaeaceae Aquilaria, (Chen xiang),* 4.35%;* Zingiberaceae Zingiber (Gan jiang),* 2.18%;* Lauraceae Cinnamomum (Rou gui),* 2.18%;* Semen Persicae (Tao ren),* 10.20%;* Pearl powder (Zhen zhu fen), *13.47% [9, 10]. JTT has long been used clinically to treat neuropsychiatric disorders. Moreover, prior studies in our laboratory have demonstrated that JTT could upregulate DAT in patients [11]. It is well known that DAT level is a standard for diagnosis of PD and can be used for evaluating therapeutic efficacy in PD treatment. Therefore, it is very interesting to investigate the effects of JTT on PD.

The neurotoxin 1-methyl-4-phenyl-1,2,3,6-tetrahydropyridine (MPTP) causes damage to dopaminergic neurons and has been widely used for generation of animal models of PD [12]. In the present study, we examined the effects of JTT on behavioral changes and characteristics of the DA system in an MPTP-induced PD mouse model. Madopar was used as a positive control drug for comparison.

## 2. Materials and Methods

### 2.1. Animals and Treatment Procedures

Male C57BL/6 mice aged 6–8 weeks (20–24 g) were randomly divided into the following groups: (1) control, (2) vehicle (distilled water), (3) high dose of JTT (0.50 g/kg/day, JTT-H), (4) middle dose of JTT (0.15 g/kg/day, JTT-M), (5) low dose of JTT (0.05 g/kg/day, JTT-L), and (6) Madopar (Roche) (0.12 g/kg/day) [13]. All groups except for the control group were given MPTP (Sigma) intraperitoneally four times at 2-hour interval at the dosage of 20 mg/kg on the 8th day of the 15-day experiment period [14]. JTT was dissolved in deionised water. The prepared suspension was intragastrically given to mice at dose of 0.50 g/kg/day, 0.15 g/kg/day, or 0.05 g/kg/day for 15 days (0.1 mL/10 g body weight). The Madopar group was given Madopar at a dose of 0.12 g/kg/day intraperitoneally for 7 days after injection of MPTP. All animals were maintained according to the international guidelines for care and use of laboratory animals. Experimental procedures involving animals were approved by the Ethics Committee of Beijing Normal University (BNU/EC/01/2011).

### 2.2. Behavioral Analyses and Brain Tissue Preparation

Six mice were randomly selected from each group and a number of behavioral tests were performed on the 9th day (1 day after MPTP) and the 15th day (7 days after MPTP).

#### 2.2.1. Locomotor Activity Test

Locomotor activity was assessed in an automated activity chamber (30 cm diameter, 15 cm high) connected to an infrared tracking analyzer that transmitted the animals' movement distance and velocity to a computer. The test was started by placing the mouse at the center of the chamber for two min for acclimatization, and then the total horizontal ambulatory route within the following 30 min was recorded by a computer analyzer.

#### 2.2.2. Rotarod Test

To determine fore limb and hind limb motor coordination and balance, a rotarod test as described previously was used [15]. Mouse was placed on the rotating bar of the rotarod unit (DXP-3; Institute of Materia Medica, Chinese Academy of Medical Sciences) set to a rotation speed of up to 18 rpm during the test session. The time spent on the rotating bar, known as the latent period, was recorded. Performance was recorded as 120 s if the latent period exceeded 120 s.

#### 2.2.3. Brain Tissue Preparation

Mice were anesthetized with sodium pentobarbital (50 mg/kg, i.p.) after the behavior test on the 15th day and rapidly perfusion fixed with saline, followed by 4% paraformaldehyde in 0.1 M phosphate buffer (pH 7.4). Then, the brains were removed and stored at −80°C until use. The frozen brains were cut into 18 *μ*m coronal sections using a cryostat microtome (CM3000; Leica, Germany). Frozen sections of the striatum and substantia nigra were used for immunohistochemistry and autoradiography experiments.

### 2.3. DAT and Tyrosine Hydroxylase (TH) Immunohistochemical Staining

Analyses of DAT immunoreactivity in the striatum and TH immunoreactivity in the substantia nigra (SN) were conducted on brain sections as previously described [16]. Briefly, sections were incubated with either rat anti-DAT (Abcam) at 1 : 1000 dilutions or mouse anti-TH (Sigma) at 1 : 10000 dilutions at 4°C overnight. Then, the slices were incubated with peroxidase-conjugated secondary antibody (ZSGB-BIO) for DAT and the VECTASTAIN ABC kit (VECTOR) for TH. All staining sections were visualized with 3,3′-diaminobenzidine tertrahydrochloride (DAB). The sections were counted using a bright-field microscope (M165FC, Leica) and optical densities (OD) of DAT and TH were calculated by Image-ProPlus software. TH-neurons were manually counted by researchers blinded to the treatment groups.

### 2.4. Autoradiography Experiments

The brains were divided into two hemispheres, one used for* in vitro *autoradiography and the other for analysis of striatal levels of DA and its metabolites [17]. Autoradiography was conducted on frozen cryostat-cut 18 *μ*m thick brain slices to measure DAT and D_2_ binding according to the procedures reported previously [18]. Briefly, brain sections were incubated for 60 min with 50 pM of [^125^I]-IBZM for D_2_ receptor (D_2_R) binding or with 50 pM of [^125^I]-*β*-CIT and in the presence of 1 mM fluoxetine for DAT binding. Nonspecific binding was determined in the adjacent slices in the presence of 10 *μ*M sulpiride (D_2_R antagonist, Sigma-Aldrich Co., USA) for D_2_R or 100 mM nomifensine (DAT antagonist, Sigma-Aldrich Co., USA) and 100 mM fluoxetine for DAT. Sections were then exposed to a super sensitive phosphor screen (PerkinElmer) for 2 h. Densitometry analysis of autoradiographs was carried out with OptiQuant software (PerkinElmer). [^125^I]-IBZM and [^125^I]-*β*-CIT were prepared according to procedures described previously [17, 19].

### 2.5. Measurement of DA and Its Metabolites in the Striatum

Levels of DA and its metabolites 3,4-dihydroxyphenylacetic acid (DOPAC) and homovanillic acid (HVA) were measured by high performance liquid chromatography with an electrochemical detector (HPLC-ECD) as previously described [20]. Briefly, the striatum was dissected, weighed, and homogenized in 0.2 M ice-cold perchloric acid. The sample was then centrifuged twice at 14,000 g for 15 min at 4°C. The supernatant was collected and filtered through a 0.22 *μ*m Millipore filter and injected into the HPLC system for analysis.

### 2.6. Statistical Analyses

Statistical analyses included one-way analysis of variance (ANOVA) followed by LSD for behavioral tests and Tukey's HSD post hoc test for all other tests. The results of immunohistochemical staining and autoradiography were indicated as a ratio compared to the control group.

## 3. Results

### 3.1. Behavioral Analyses

#### 3.1.1. Locomotor Activity

**Figure 1:**
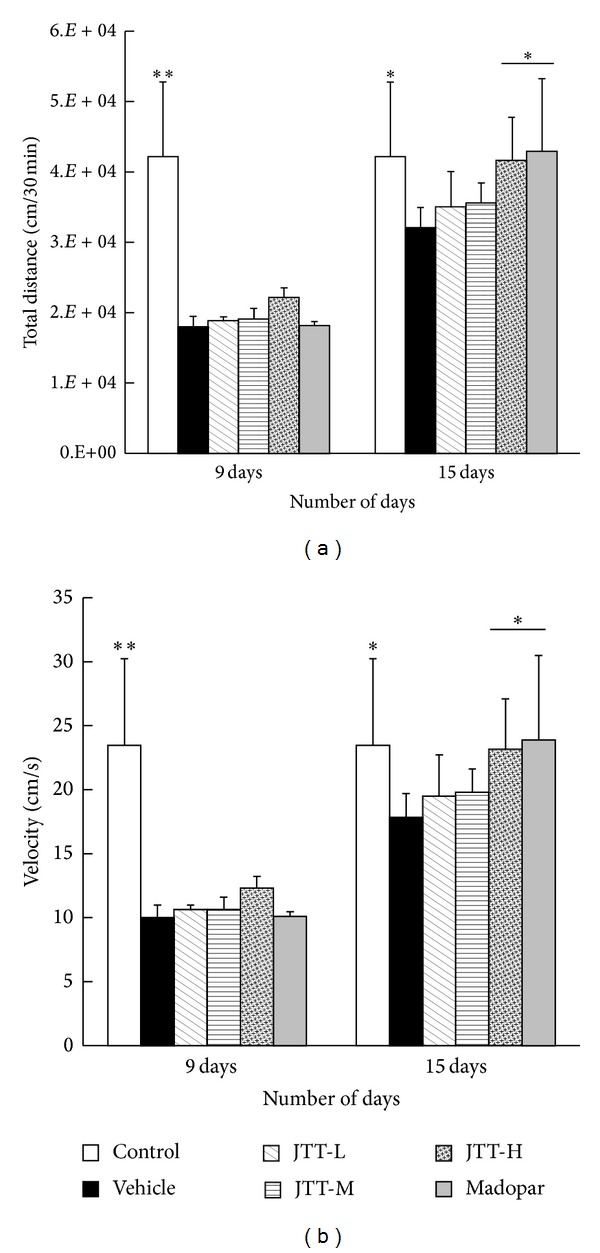
JTT improved MPTP-induced locomotor activity deficits in PD mice. These tests were used to assess mouse behaviors on the 9th day (1 day after injection of MPTP) and 15th day (7 days after injection of MPTP). All the data, total movement distance (a), and mean velocity (b), *n* = 4–6, were shown as mean ± S.E.M, *P < 0.05, **P < 0.01 versus vehicle group.

Locomotor activity testing is a commonly used technique for behavioral monitoring in PD animal models. In this study, it was used to evaluate motor impairment in the MPTP-treated animals. As shown in Figures 1(a) and 1(b), on the 9th day, all animals in MPTP-treated groups exhibited a significant reduction in total movement distance and mean velocity compared to those in the control group (*P* < 0.01). On the 15th day, there was a marked improvement in locomotor activity in mice treated with high dose JTT (JTT-H) compared to those treated with the vehicle, including both movement distance and mean velocity (*P* < 0.05). The group of mice treated with Madopar also demonstrated significant improvements compared with the vehicle group in movement distance and mean velocity (*P* < 0.01).

#### 3.1.2. Rotarod Test

**Figure 2:**
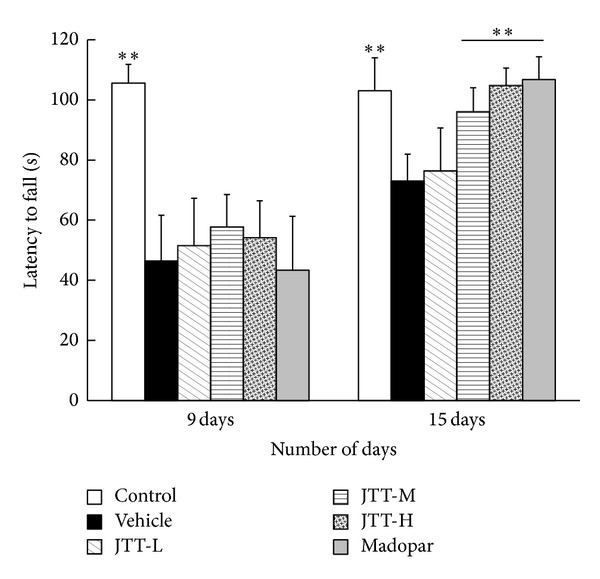
JTT improved MPTP-induced latent period deficit in PD mice. Test was done on the 9th day (1 day after injection of MPTP) and 15th day (7 days after injection of MPTP). The data latent period, *n* = 8–10, were shown as mean ± S.E.M, *P < 0.05, **P < 0.01 versus vehicle group.

The restorative effects of JTT on MPTP-induced motor coordination deficiencies were tested using the rotarod test. As shown in Figure 2, on the 9th day, all mice injected with MPTP had a significant reduction in rotarod performance compared to control mice (*P* < 0.01). On the 15th day, a significant difference was observed between control and vehicle (*P* < 0.01) mice. The latent period significantly increased in the JTT-H and JTT-M groups (*P* < 0.01) and the Madopar group (*P* < 0.01) when compared with mice in the vehicle group. JTT effectively countered the MPTP-induced behavioral coordination deficiencies in a dose-dependent manner.

### 3.2. Effects of JTT on MPTP-Induced Decreases in TH Positive Neurons in the SN

**Figure 3:**
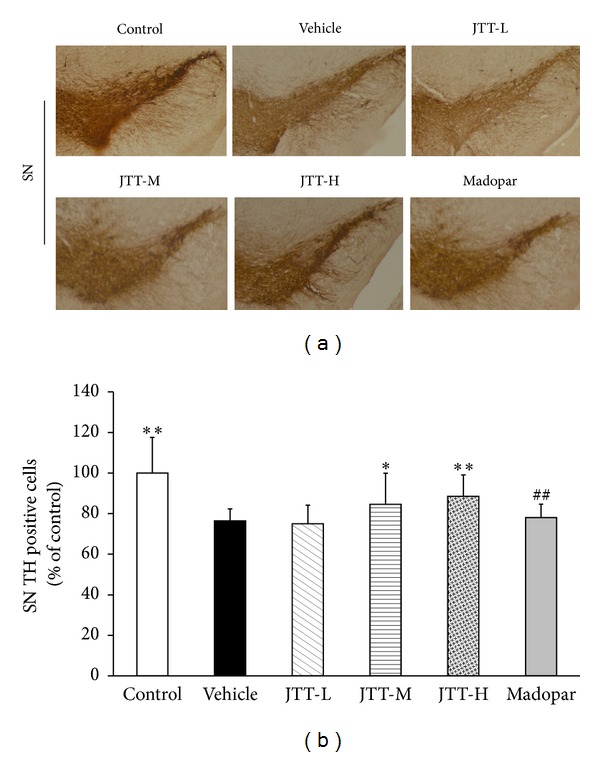
JTT protected against MPTP-induced reductions in TH expression in the mouse SN. C57BL/6 mice were treated with JTT for 7 days and then MPTP (20 mg/kg, 4 times, at 2-hour interval, i.p.) was injected on the 8th day. Mice were sacrificed on the 7th day after MPTP injection (15th day of the experiment period) and after behavioral tests. Photomicrographs of representative SN (a) sections stained with an antibody against TH. Reduced activity of TH-neurons was observed in MPTP treated mice, which was partially prevented by treatment with JTT. The number of TH-positive neurons in the SN (b) was expressed as the mean ± S.D, *n* = 5. *P < 0.05, **P < 0.01 versus vehicle group, ^##^ *P* < 0.01 versus JTT-H.

Immunohistochemical staining was used to measure the levels of TH in the SN. As shown in Figures 3(a) and 3(b), MPTP exposure led to a remarkable loss of TH positive neurons in the SN (*P* < 0.01) compared to control mice. Treatment with JTT-H and JTT-M significantly halted TH-neuron reductions (JTT-H, *P* < 0.01; JTT-M, *P* < 0.05), while treatment with Madopar had no effect. This result showed that JTT significantly protected against MPTP-induced reduction in TH-neurons in the mouse SN while Madopar did not.

### 3.3. DAT in the Striatum Measured by Immunohistochemical Staining and Autoradiography

**Figure 4:**
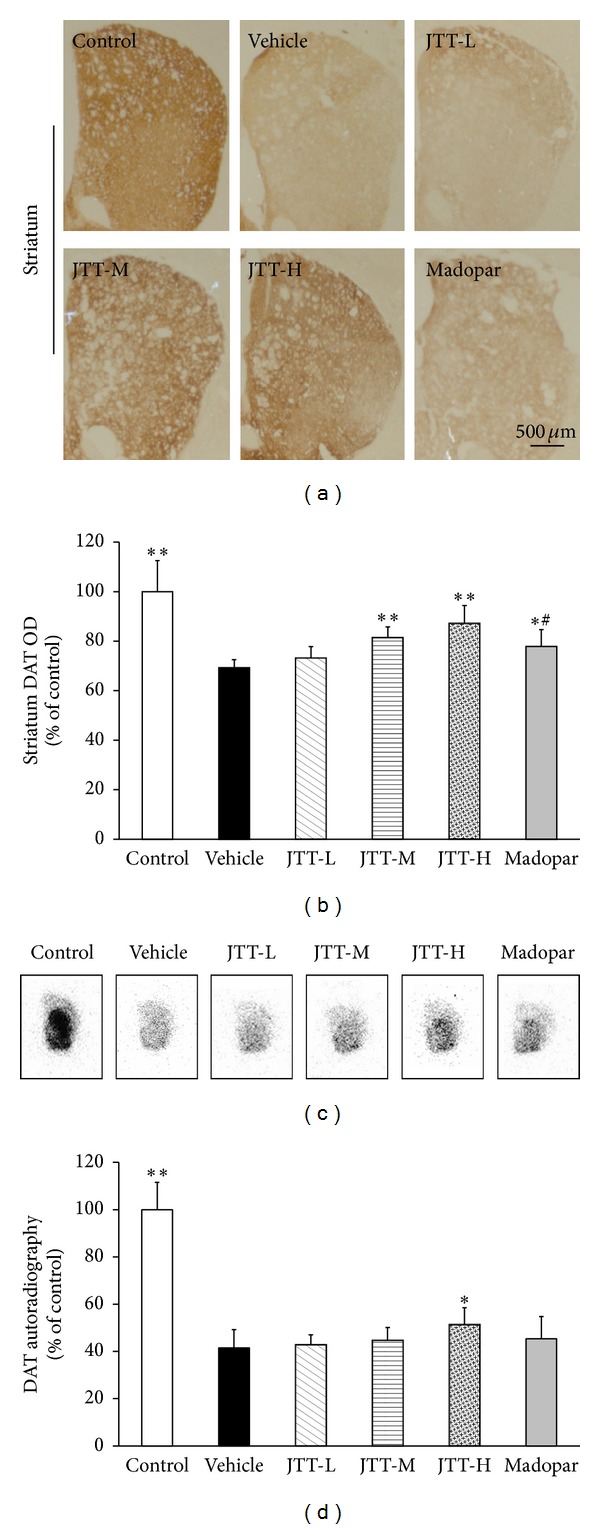
JTT protected against MPTP-induced reductions in DAT levels of the mouse striatum as measured by immunohistochemistry and autoradiography. MPTP (20 mg/kg, 4 times, at 2-hour interval, i.p.) was injected on the 8th day of the 15-day experiment period. The animals were sacrificed on the 7th day after MPTP injections for immunohistochemistry and autoradiography studies. Photomicrographs of representative striatum immunohistochemistry sections (a) stained with a DAT antibody. Autoradiography of DAT binding using [^125^I]-*β*-CIT (c). A decrease in DAT levels was observed in MPTP mice. Treatment with JTT partially prevented this decrease. Optical density of DAT-positive fibers in the striatum (b), radioactivity binding of DAT in striatum (d) are expressed as the mean ± S.D, *n* = 5. *P < 0.05, **P < 0.01 versus vehicle group, ^#^ *P* < 0.05 versus JTT-H.

DAT is a key protein used in the clinical diagnosis of PD. In this study, immunochemical staining and autoradiography were used to detect DAT levels in the striatum. As shown in Figures 4(a) and 4(b), MPTP treatment elicited a significant reduction in DAT levels in the striatum (*P* < 0.01). Treatment with JTT-H (*P* < 0.01) and JTT-M (*P* < 0.05) significantly protected against this reduction. A comparison between JTT and Madopar found that JTT-H was significantly superior in its protective/restorative effect (*P* < 0.05): DAT level increased by 18% in the JTT-H group, but only by 9% in the Madopar group when compared to the vehicle group.

Figures 4(c) and 4(d) illustrate the range of DAT levels after drug treatments via autoradiography using [^125^I]-*β*-CIT. MPTP treatment led to a significant reduction in DAT binding in the striatum compared with control mice. This reduction was attenuated in mice treated with JTT, while treatment with Madopar showed no significant effect when compared to vehicle mice. These results indicate that JTT could significantly protect against MPTP-induced reductions in DAT levels in the mouse striatum and that JTT-H is more effective than Madopar.

### 3.4. Autoradiography Detected D_2_R in Striatum

**Figure 5:**
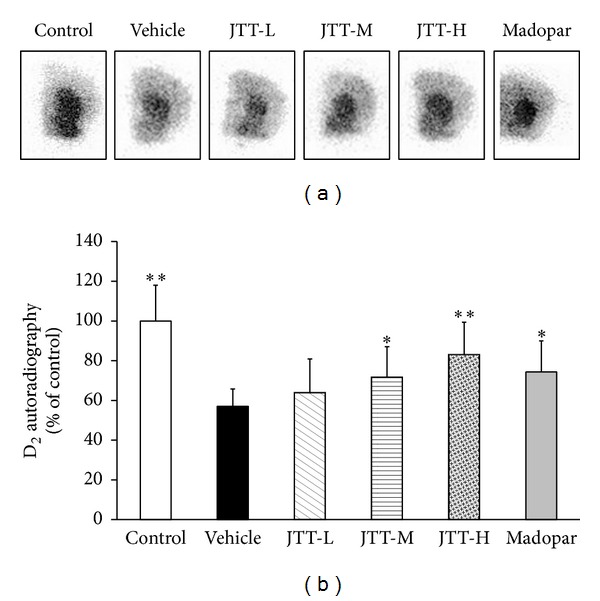
JTT protected against MPTP-induced reductions in D_2_R expression in the mouse striatum as measured by autoradiography. MPTP (20 mg/kg, 4 times, at 2-hour interval, i.p.) was injected on the 8th day of the 15-day experiment period. Mice were sacrificed for the autoradiography study on the 7th day after MPTP injections. [^125^I]-IBZM D_2_ binding is shown in autoradiograms (a). A decrease in D_2_R expression was observed in MPTP mice. Treatment with JTT partially prevented this decrease. Radioactivity binding of D_2_ in the striatum (b) is expressed as the mean ± S.D, *n* = 5. *P < 0.05, **P < 0.01 versus vehicle group.

DA receptors gradually decrease in the progression of PD over time and the D_2_R is one of the main receptors of the DA system. Autoradiography with [^125^I]-IBZM was used to detect levels of D_2_R expression in the striatum. As shown in Figure 5, MPTP treatment elicited a significant reduction in D_2_ binding with [^125^I]-IBZM in striatum compared with that of control mice (*P* < 0.01). This reduction in striatal D_2_R levels was attenuated in mice treated with JTT-H and JTT-M in a dose-dependent manner (JTT-H, *P* < 0.01; JTT-M, *P* < 0.05). Although Madopar also had a significant effect to increase D_2_ levels (*P* < 0.05) in MPTP-treated animals, JTT-H had a stronger effect: JTT-H reversed 26% of the MPTP-induced reductions in D_2_ binding, compared with 17% by Madopar.

### 3.5. Levels of DA and Its Metabolites in the Striatum

**Figure 6:**
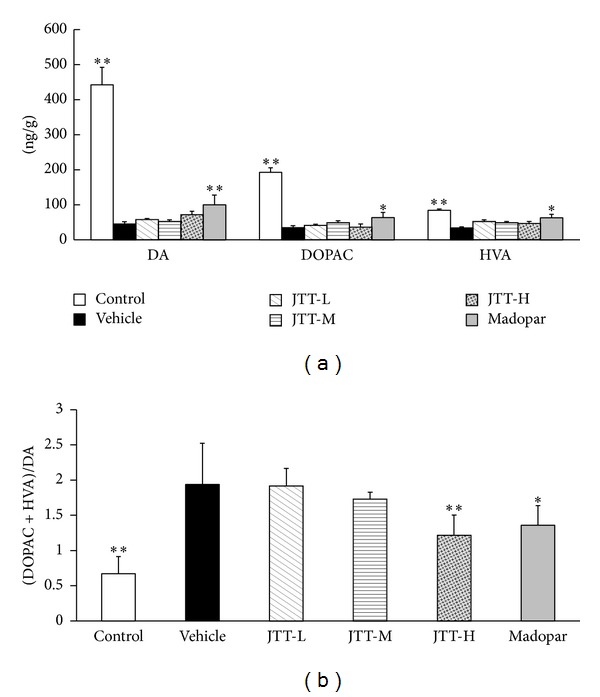
JTT inhibited the increase in the ratio of DOPAC and HVA to DA in the striatum of MPTP mice. MPTP (20 mg/kg, 4 times, at 2-hour interval, i.p.) was injected on the 8th day of 15-day experiment period. The contents of DA, DOPAC, and HVA were detected by HPLC-ECD and the ratio of (DOPAC + HVA) to DA was calculated. Data regarding DA, DOPAC, HVA (a), and (DOPAC + HVA)/DA ratios (b) are expressed as the mean ± S.D, *n* = 4-5. *P < 0.05, **P < 0.01 versus vehicle group.

The levels of DA and its metabolites in the striatum were measured by HPLC-ECD. As shown in Figure 6(a), mice in the MPTP vehicle group showed a remarkable depletion of DA, DOPAC, and HVA in the striatum compared with those in the control group (*P* < 0.01). The ratio of DOPAC and HVA to DA (Figure 6(b)) was significantly higher (*P* < 0.01) in the vehicle group than the control, which is indicative of increased DA metabolism in the vehicle group. Treatment with JTT effectively inhibited the increase in the DOPAC and HVA to DA ratio (JTT-H, *P* < 0.01) in a dose-dependent fashion, despite the fact that the three dosages of JTT did not significantly alter striatal levels of DA and its metabolites. The effect of inhibiting this increased ratio was stronger with JTT-H and JTT-M than with Madopar (*P* < 0.05). However, Madopar appeared to increase the levels of DA, DOPAC, and HVA in the striatum (*P* < 0.05).

## 4. Discussion

The present study investigated the ability of JTT to offer protection against the neurotoxicity of MPTP. Our findings indicated that high dose of JTT (JTT-H, 0.50 g/kg/day) effectively protected against the MPTP-induced deficiencies in behavioral activities, in an efficacy equal to that of Madopar. Moreover, we found that JTT could protect against the loss of TH-neurons in the SN. JTT also attenuated the reduction in DAT levels in the striatum induced by MPTP. Furthermore, JTT protected against the reduction in striatum D_2_ levels typically induced by MPTP in a dose-dependent manner. We also found that JTT could attenuate the increase in DA metabolism rates in MPTP-treated mice. In general, these protective/restorative effects were stronger in animal treated with JTT-H than Madopar.

Movement deficits in PD result from dopaminergic dysfunction in the brain. In our study, JTT treatment clearly did improve MPTP-induced motor deficits. Notably, we found that JTT could inhibit the rate of DA metabolism while failing to significantly increase the concentrations of DA and its metabolites. One explanation for these findings may be that behavioral changes are not solely directly related to striatal DA levels, and multiple factors are likely to be at play [21]. As is well known, long-term treatment with Madopar induces many side-effects and these side-effects coincide with the rise and fall of DA levels in the brain that occur in the course of L-DOPA therapy [22]. As for JTT, preclinical toxicological studies have found no apparent toxicity even at high doses (e.g., 9.8 g/kg/day) in SD rats during long-term use (6 months) [23]. In the clinical setting JTT also demonstrated no obvious adverse effects [24–26]. In the present study, JTT was found to improve striatal DA levels only appreciably but effectively attenuate the increase in DA turnover rate. This may suggest that long-term treatment with JTT is effective and will not induce the same side-effects as Madopar.

PD is characterized by degeneration of DA neurons and corresponds to loss of DAT, a membrane protein important for DA reuptake. Decreasing DAT levels is the gold standard by which clinical diagnoses of PD are made using medical imaging methods [27, 28]. In the present study, we found that JTT protected against MPTP-induced DAT reduction in a dose-dependent manner. Relatively speaking, Madopar treatment has little effect on DAT levels. It has been reported that ginseng, which is an active ingredient found in JTT, can significantly preserve DAT levels in the striatum [29]. Changes observed in DAT levels during JTT treatment may mainly be due to its protection of DA turnover, but may also result from the direct action of JTT on DAT expression.

The D_2_R is one of the key receptors associated with PD but remains controversial because levels of D_2_R expression may decrease in PD patients or not. Antonini et al. found a significant reduction in putamen D_2_ binding to [^11^C]raclopride in PD patients who had carried a diagnosis for 3–5 years [30]. Rinne et al. found that striatal D_2_ increased in early PD patients [31]. In this study, we found significant decline in striatal D_2_ binding to [^125^I]-IBZM in MPTP-treated mice. Meanwhile, JTT significantly inhibited this reduction, suggesting that JTT may play a role in reversing D_2_R decline, which may be beneficial to the treatment of PD.

In order to investigate the neuroprotective effect of JTT in MPTP-induced PD model, we treated mice with JTT before MPTP, in a paradigm similar to that used in other studies reviewed by More et al. [7]. JTT was found to inhibit the reduction in positive TH-neurons in SN typically observed in vehicle MPTP mice. This suggests that JTT might offer protection against dopaminergic neurodegeneration induced by the neurotoxicity of MPTP. Moreover, JTT upregulates the levels of DAT and D_2_R in the striatum. Some components in the JTT prescription have been reported to have protective effects on neurons and upregulate DA tone. For example,* Radix salvia* and* Radix ginseng*, which are components in JTT, have been reported to be effective in protecting neurons from neurotoxicity [32–34]. Some bioactive compounds from herbal medicine have been reported to provide a significant neuroprotective effect in PD models and JTT does contain many constituents with neuroprotective effect, such as ginsenoside Rg1 [35]. Scopolamine, which is an active ingredient in JTT, is effective at enhancing DAT availability through the inhibition of muscarinic cholinergic neuronal activity [36]. The mechanism underlying the ability of the JTT to treat MPTP-induced PD mice, even though not entirely clear, is likely to derive from the combined effects of its active ingredients. JTT may act on multiple targets, which reflects the advantage of TCM.

## 5. Conclusions 

Evidence from the present study appears to indicate that JTT can play an important role in behavioral and motor function improvement in MPTP-induced PD model mice. This is related to the DA system, including DAT, D_2_R, and TH. Therefore, JTT may be considered as a potential complementary therapeutic agent for PD treatment, because it not only provides relief of motor symptoms, but also protects neurons and involves the DA system to restore levels of DAT and D_2_R.
